# Introducing the Stool Stomper: A Device Designed to Enable Accelerated and Standardized Stool Sample Preparation Using the Kato–Katz Technique

**DOI:** 10.3390/bioengineering12040432

**Published:** 2025-04-19

**Authors:** Asher C. Altman, Andrew D. Bohner, Victoria J. Brown, Lauren Barger, Rudolph L. Gleason, James K. Rains, James B. Stubbs, Kelsey P. Kubelick, Mariana Stephens

**Affiliations:** 1 Wallace H. Coulter Department of Biomedical Engineering, Georgia Institute of Technology and Emory, University School of Medicine, Atlanta, GA 30332, USA; asheraltman@gatech.edu (A.C.A.); bohner49@rowan.edu (A.D.B.); rudy.gleason@me.gatech.edu (R.L.G.);; 2 School of Chemical and Biomolecular Engineering, Georgia Institute of Technology, Atlanta, GA 30332, USA; 3 George W. Woodruff School of Mechanical Engineering, Georgia Institute of Technology, Atlanta, GA 30332, USA; 4 School of Electrical and Computer Engineering, Georgia Institute of Technology, Atlanta, GA 30332, USA; 5 Children Without Worms, The Task Force for Global Health, Atlanta, GA 30030, USA

**Keywords:** Kato–Katz technique, human-centered device design, soil-transmitted helminths, neglected tropical diseases, global health

## Abstract

Soil-transmitted helminths (STHs) are parasitic worms that impact over 1.5 billion people globally. The Kato–Katz technique analyzes stool samples for STHs, allowing for individual diagnoses of STH infection and the estimation of community-level prevalence. One challenge that arises with the procedure is that lab technicians often struggle to prepare microscope slides of sufficient quality for analysis after one attempt. As a result, Kato–Katz procedures are repeated, wasting time and resources. To aid technicians during in-field slide preparation, we created the Stool Stomper. The Stool Stomper is a user-friendly, handheld mechanical device that applies constant, uniform pressure to stool samples to ensure standardized sample preparation onto microscope slides to improve egg counts. The Stool Stomper was assessed using artificial eggs during in-country testing in a lab setting in Dodoma, Tanzania, by lab technicians with various experience levels, from beginner to advanced. Compared to the traditional method, we found that the Stool Stomper reduced slide preparation time, reduced artificial egg counting time, and standardized artificial egg counts with more consistent and accurate readings. The current pilot study highlights the potential for future development and integration of the Stool Stomper device into the Kato–Katz technique to improve community-based STH treatment.

## 1. Introduction

Soil-transmitted helminths (STHs) are parasitic worms that impact over 1.5 billion people globally, affecting one out of every five persons [1,2,3]. These conditions are neglected tropical diseases (NTDs) that primarily affect Sub-Saharan Africa and tropical Asia [4]. STHs are typically transmitted through oral contact with infected feces in areas with poor hygiene practices by exposure to egg-contaminated food, water sources, or soil [5]. The primary method of treatment is preventative oral chemotherapy, such as Albendazole and Mebendazole, distributed on a community level by nongovernmental organizations or public health entities [6,7]. High infection levels can cause significant morbidities. Low infection levels are linked to diarrhea, severe dysentery, anemia, and halted physical and cognitive development in children [8,9]. Therefore, STHs impose a significant burden on developing nations [10].

**Figure 1:**
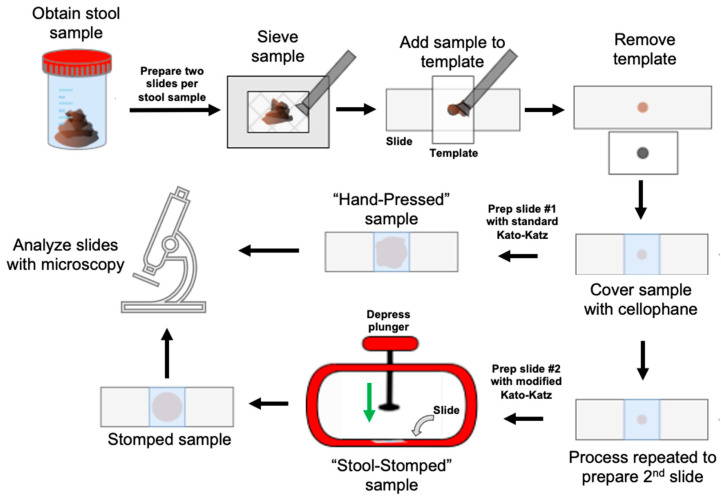
Schematic of the Kato–Katz protocol with the addition of the Stool Stomper. Lab technicians follow the standard Kato–Katz procedure to prepare stool samples through the step of covering the stool sample with cellophane. At this point, rather than the “hand-pressed” preparation of the sample onto the microscope slide, the “Stool Stomper” is introduced to uniformly, evenly, and consistently distribute the stool sample onto the slide via a plunger mechanism to improve downstream microscopic analysis.

The World Health Organization recommends the Kato–Katz technique for preparing and analyzing stool samples for identifying STHs [7]. The Kato–Katz technique, depicted in Figure 1, was created in 1954 to measure an individual’s egg burden through the microscopic analysis of a provided fecal sample [11]. The manual collection, preparation, and evaluation of stool sample slides is performed by lab technicians, who are often working with mobile lab equipment in rural areas that lack basic resources, such as electricity. Across a community-wide survey, egg burdens inform the allocation of preventative oral chemotherapy in heavily affected areas, allowing for population-based control of NTDs.

The Kato–Katz technique is not without its flaws. The technique lacks inter-technician and intra-technician reliability and reading acuity, is time-consuming, and consistently under-represents egg burden [12,13,14,15]. The inconsistency in sample preparation and reading acuity is linked to inherent issues with the technique and training of lab technicians. Regarding the Kato–Katz protocol, stool samples must be evenly flattened to display all eggs present, and samples must be read within an hour of preparation to prevent hookworm egg degradation [16]. Achieving these goals can be difficult and cumbersome in a field setting due to inconsistent working conditions and a large accumulation of prepared samples, overwhelming egg-reading personnel. Labs are often understaffed and must rely on volunteers or auxiliary employees from the community to help prepare stool sample microscope slides. These additional workers lack experience with performing the Kato–Katz technique, further compounding errors. In turn, more experienced lab technicians frequently need to instruct auxiliary personnel, and the Kato–Katz procedure is repeated on a given sample until the thick smears can be interpreted. Clearly, there is a critical need for a robust improvement to the current protocol. Several techniques and devices have been implemented to improve on the Kato–Katz protocol, such as the mini-FLOTAC and the FlukeFinder [17,18]. Although both methods outperform the classic Kato–Katz technique, we propose an alternative approach that builds upon the existing health infrastructure and more seamlessly integrates with current sample preparation protocols.

Herein, we created the “Stool Stomper”—a reusable device designed to augment the standard Kato–Katz technique to improve the sample preparation and diagnosis of STH. In the classic Kato–Katz technique, lab technicians manually compress a stool sample onto a microscope slide with their fingers. In contrast, the Stool Stomper utilizes a plunger with a stop to evenly press the stool sample (Figure 1).

Our study has two major goals to assess the utility of the Stool Stomper and its benefit to the Kato–Katz technique. The first goal of our study is to demonstrate that the Stool Stomper improves intra- and inter-lab technician reading accuracy by evenly distributing stool samples across a microscope slide and reducing egg clustering, where we hypothesize that more consistent sample preparation reduces variability in egg counts. The second goal of our study is to assess the impact of the Stool Stomper on the time required to prepare and read microscope slides. We hypothesize that standardizing slide preparation will reduce preparation time, read time, and minimize delays caused by slides of insufficient quality, requiring repeat sample prep. The current results support incorporation of the Stool Stomper into the Kato–Katz technique, highlighting the potential of the device to improve reading accuracy and reducing sample preparation time.

## 2. Materials and Methods

### 2.1. Design

The Stool Stomper device was designed via a human-centered design process involving over 20 stakeholder interviews with users and global experts to define key needs for standardizing stool sample preparation in the Kato–Katz technique. Design requirements included: (1) accurate function in natural light for field/low-resource laboratories; (2) reduced slide preparation and reading time; (3) low production cost; and (4) portability. Functional accuracy was prioritized to ensure consistency across technicians. From one hundred concepts, the “Stool Stomper” emerged as a simple, handheld mechanical device using a plunger and tension spring to apply consistent pressure, enabling uniform sample spreading for improved microscopic analysis.

**Figure 2:**
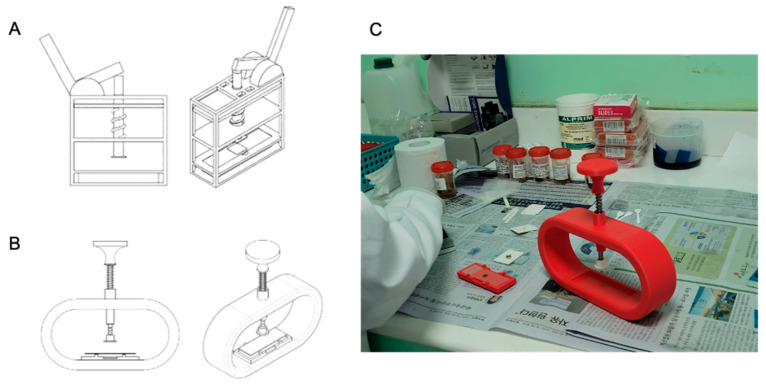
The Stool Stomper device. (**A**) The Stool Stomper’s first iteration utilized a lever press mechanism. (**B**) The current version of the Stool Stomper utilizes a plunger push–press mechanism. The plunger is built to permit 20 N of force applied to a slide, causing stool samples to be spread evenly in a 14 mm diameter circle. (**C**) Photograph of the Stool Stomper in use by a lab technician in Dodoma, Tanzania.

The final design of the Stool Stomper (Figure 2) consisted of a six-component structure: a main body, slide carriage, plunger top, tension spring, steel rod, and plunger bottom. The tension spring and steel rod were designed to deliver 20 N of force across an area of approximately 14 mm in diameter, similar to the force used in hand-pressed samples, to not crush hookworm eggs. The device was primarily built via 3D printing. Polylactic acid (PLA) was used to print the main body and slide carriage, taking eight hours and one hour, respectively. The plunger top and bottom were printed out of acrylonitrile butadiene styrene (ABS), which had a combined print time of one hour. The total prototype device construction time was approximately 11 h.

The Stool Stomper underwent iterative design refinements to optimize durability and usability. Initially featuring a lever-based pressing mechanism (Figure 2A), feedback revealed inconsistent performance on uneven surfaces, prompting a switch to a plunger system that also reduced part count. A 6-foot drop test confirmed body durability but revealed spring-plunger detachment when under stress, leading to a shortened plunger with a curved top for secure C-ring attachment. The original slide carriage hindered rapid slide changes, so a removable carriage was implemented to improve accessibility and cleaning efficiency.

### 2.2. Operator Screening

The study was conducted at the Benjamin Mkapa Hospital (Hospitali ya Benjamin Mkapa) in Dodoma, Tanzania. The facility contains a diagnostic lab staffed by technicians for processing stool samples and assessing STH via microscopic analysis. These lab technicians were asked to report their relative skill levels in performing the Kato–Katz procedure, stating the number of years of direct experience executing the procedure. Technicians were then sorted into “Beginner”, “Intermediate”, or “Advanced” groups based on their years of hands-on experience. “Beginner” was defined as a technician who was new to the Kato–Katz technique. “Intermediate” was defined as a technician who had carried out the Kato–Katz technique for 6 months to 9 years. “Advanced” was defined as a technician with 10+ years of experience using the Kato–Katz technique. In total, the study consisted of two “Advanced”, two “Intermediate”, and one “Beginner” technician.

### 2.3. Study Design and Technician Blinding

**Figure 3:**
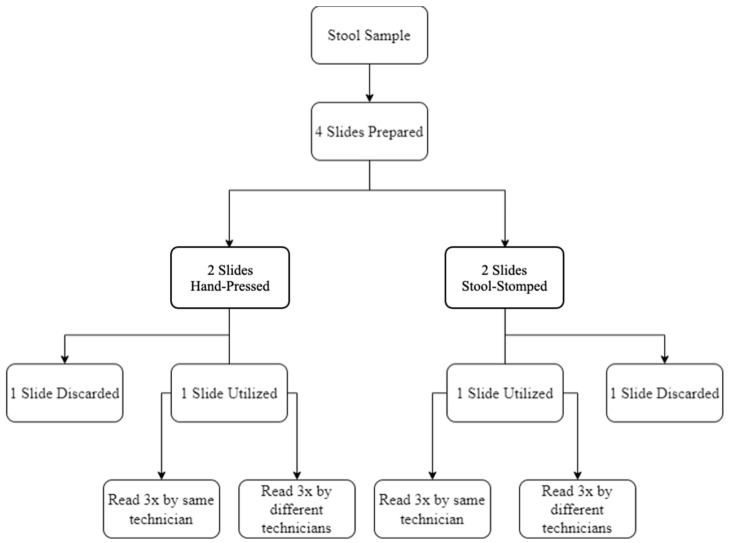
Diagram of the sample and slide preparation study workflow. For each stool sample, four slides were prepared in total—two via the traditional hand-pressed approach and two via the Stool Stomper approach. Excess slides were kept throughout the study to blind lab technicians and create the appearance of a larger reading pool.

A blinded comparative study evaluated stool sample preparation using the Stool Stomper versus traditional hand-pressed prepared slides, analyzing reproducibility, reading acuity, and preparation/reading time. Both methods followed the Kato–Katz technique. However, the Stool Stomper replaced less controllable hand-pressing with a standardized plunger mechanism. For each sample, four slides were prepared (two per method) and each slide was read six times in total by four different technicians (Figure 3). Sample preparation is described further in the “Stool Sample Preparation” section below.

The study employed multiple blinding techniques to minimize bias. Slides were standardized by eliminating plastic overhang and debris. For each sample, four slides were prepared (two read, two reserved) to obscure slide identity during analysis. An obfuscated labeling system used faux slide labels, with incremental slide rotations of 180-degrees between readings to alter appearance. Technicians received slides in batches of four, unaware that each slide was read three times by themselves and three times by others. All labels were tracked to record technician proficiency, egg counts, and processing times, while maintaining blinding throughout.

### 2.4. Stool Sample Preparation

**Figure 4:**
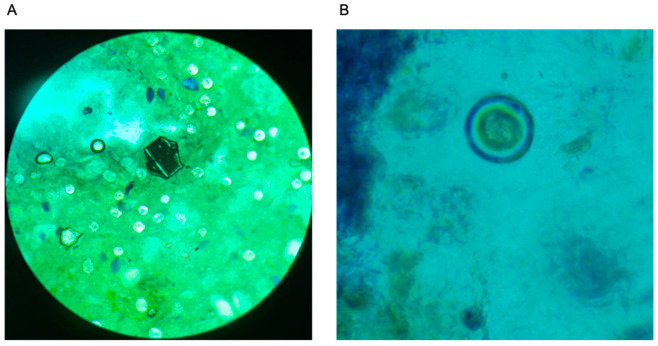
Brightfield micrographs of Stool Samples. (**A**) A 40× view of one grain of shape-cut craft glitter under compound light microscopy. The distinct hexagonal nature of shape-cut glitter allowed technicians to distinguish artificial eggs from background detritus when reading samples, creating a controlled, reproducible test scenario. (**B**) A 40× view of one *Taenia Solium* tapeworm egg in an egg-spiked sample.

During the on-site field study in Dodoma, Tanzania, negligible STH egg burden was observed in stool samples provided by the Ministry of Health. To proceed with our studies within the limited time on site, provided stool samples were modified by adding “artificial egg” substitutes. A variety of substitutes were recommended by experts in the STH field, namely shape-cut craft glitter, crushed graphite, ground spices, or egg-spiking samples with a separate source of isolated STH eggs. Egg-spiking was not feasible due to the limited number of STH eggs on-site at the lab. Rigorous microscopic analysis was carried out to assess which STH egg substitute possessed similar properties to real STH eggs. Shape-cut craft glitter was selected for the artificial eggs due to its uniform presentation when reading, repeatability for sample preparation, and similar size to natural eggs (Figure 4). Upon handling and analyzing the glitter-spiked samples, advanced-level lab technicians confirmed that slide preparation and egg counting were near-identical to slides with STH eggs, making the spiked samples a controlled, appropriate proxy for device testing. While this limitation is regrettable, it is important to note the value of the acquired lab technician artificial egg data to inform device design and further development. Approximately 0.1 g of glitter was added to each stool sample to mimic low-burden egg samples. The allocated glitter amount was uniformly mixed in the stool sample using a plastic spatula.

### 2.5. Slide Preparation Study

Technicians received brief hands-on training with the Stool Stomper, demonstrating its integration into the latter steps of the Kato–Katz technique (Figure 1). After observing proper use, the study team corrected handling errors before testing commenced.

Following standard protocol, methylene blue-soaked cling wrap was prepared in advance. Technicians began slide preparation using the standard initial steps of the Kato–Katz technique, completing the sample processing with either the hand-pressed or the Stool Stomper method while being timed. Timing started when the technician picked up the stool sample vessel and stopped once two slides were prepared. Notably, some technicians recorded preparation times rounded to the nearest minute, causing data clustering. Prepared slides were subsequently distributed to a separate group of reading technicians for egg count analysis.

### 2.6. Egg Counting Study

Reading technicians were assigned to individual stations with a compound light microscope (Optika B-510ASB Phase Contrast Microscope, Optika Microscopes, Ponteranica, Italy) equipped with 10× and 40× magnification, a handheld tally counter, and a survey sheet to record the faux slide label, number of eggs, and lab technician experience level. A study team member timed each reading technician as they counted the eggs with the hand tally. The timer began when the slide was loaded onto the microscope stage and ended once the technician recorded the egg count for the slide on their survey sheet.

### 2.7. Statistical Analysis

Data were assessed using Excel 16.0 and Python 3 on Jupyter Notebook 6.4.12 through Anaconda Navigator 2.5.1.

Prior to beginning the study, an advanced-level technician determined the average egg count for each sample, which served as the gold standard or ground truth for sample preparation and statistical analysis. Results from intermediate- and beginner-level technicians were compared to the advanced-level technician’s ground truth to evaluate acuity and reproducibility for both methods of preparation (hand-pressed or stool-stomped). Reading acuity was defined as a greater number of eggs counted by technicians because the Kato–Katz technique inherently underestimates egg counts [19].

A variance analysis to compare the spread of egg counts was carried out using the grand mean of each method of preparation’s standard deviations. A 95% confidence interval was found for this grand mean. Grand means were calculated assuming equal weighting due to a uniform sub-sampling size.

A one-tailed left-sided paired *t*-test was performed using an α of 0.05 for each sample comparing the differences in egg count of each read and the gold standard across both preparation methods. This same test was used for comparison of the sample distributions by method of preparation. A priori power analysis was conducted to determine that 12 samples were needed with an effect size of 0.8 to achieve a power of 0.83 for the one-tailed left sided *t*-test. Missing technician egg counts for the first and second samples (in the first series of 4 slides) of egg counting testing were filled by averaging all technician readings for those samples to permit statistical testing. Where necessary, an average value across all utilized readings was substituted as a difference placeholder for missing egg counts to allow for statistical testing. This substitution occurred in stool-stomped samples 1 and 2 (2× each) and stool-stomped samples 4, 7, and 12 (1× each).

## 3. Results

**Figure 5:**
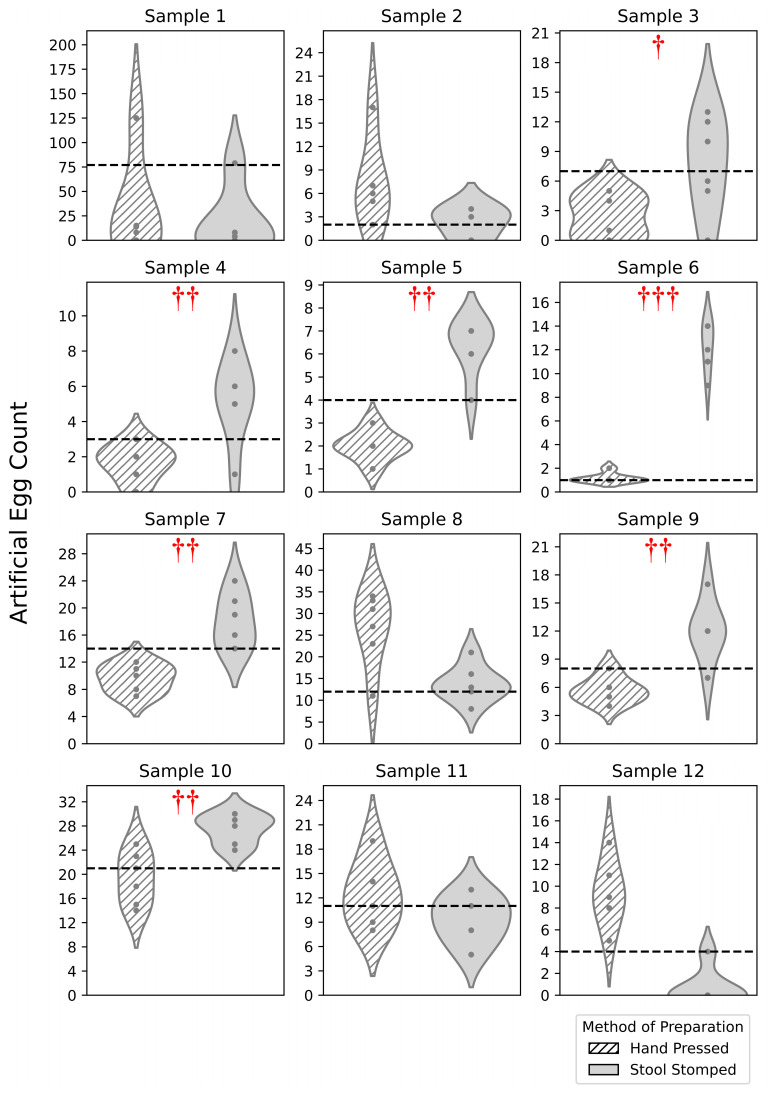
Comparison of reading acuity for each method of preparation on a per-sample basis for 12 fecal samples. The dashed lines indicate the gold standard egg count, or ground truth, for each sample determined by a weighted average of readings from advanced-level technicians. Daggers denote significance level for a one-tailed *t*-test comparing the differences in the means of the egg count for each preparation method. The study employed significance thresholds of 0.05, 0.01, and 0.001, denoted with red daggers †, ††, and †††, respectively.

The goal of this assessment was to assess the Stool Stomper method in terms of reading uniformity, slide preparation time, and egg counting time. Figure 5 depicts differences in performance for each method of preparation as measured by egg sample reading uniformity. The results for each sample compile all technician readings with no distinction between experience levels. Notably, stool-stomped samples have a lower count dispersal compared to hand-pressed samples. Therefore, the acuity of stool-stomped samples improved with lower dispersal across a lower range of egg counts in prepared samples. The sample-separated, wider violin plots of the stool-stomped samples correspond to a 95% confidence interval of 11.48 ± 2.70 eggs. The hand-pressed samples correspond to a 95% confidence interval of 10.58 ± 3.92 eggs. The reduced spread of the confidence interval for the Stool Stomper of 2.70 eggs is indicative of higher reliability in egg count across lab technicians and supports the device’s potential for in-field work.

A left-sided one-tailed *t*-test was then performed comparing the means for each sample across the two preparation methods. A statistically significant increase in the mean egg count was observed in seven samples prepared via the stool-stomped method. Results indicate a comparatively improved reading acuity of the Stool Stomper, meaning that more eggs were found when reading the same stool sample.

**Figure 6:**
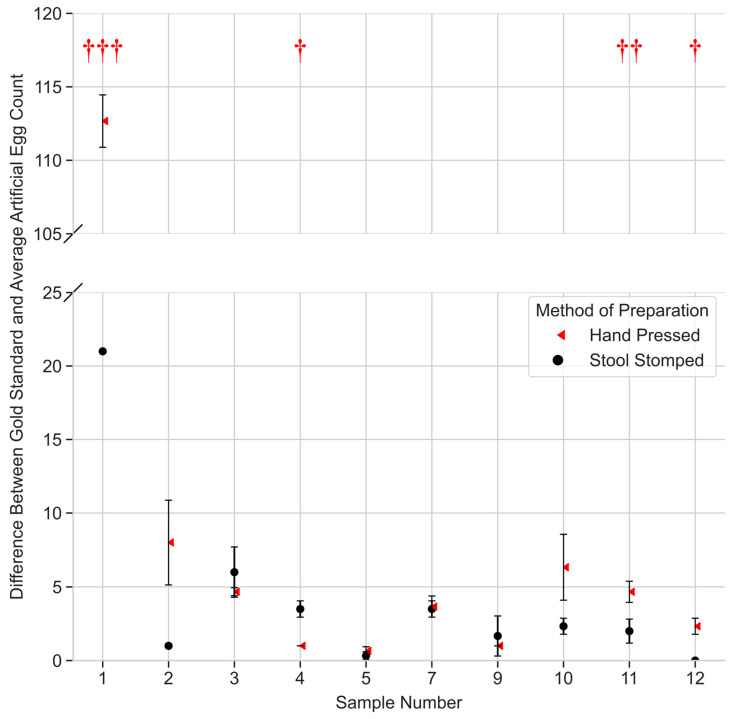
Analysis of differences in egg counts for each preparation method compared to the gold standard. Each data point represents the difference between the average egg count across all reads from technicians with different experience levels compared to the ground truth. Note the *y*-axis bridge between 25 and 110 due to the large difference in hand-pressed samples observed solely in Sample 1 readings. Hand-pressed preparation is denoted by red triangles. Stool Stomper preparation is denoted by black circles. Error bars represent the 95% confidence interval. Daggers denote significance level for a one-tailed *t*-test, comparing the differences between the ground truth and each method of preparation. The study employed significance thresholds of 0.05, 0.01, and 0.001, denoted with red daggers †, ††, and †††, respectively.

The average egg counts for each sample and preparation method, stool-stomped or hand-pressed, were then compared to the gold standard count (Figure 6). In this case, the average egg count still captures the reads from all technicians, regardless of experience level. Samples 6 and 8 were not included for this portion of the study due to limited advanced technician data available for difference comparison. 95% CIs for the difference between the gold standard count were found to be 4.13 ± 2.29 eggs when using the Stool Stomper and 14.5 ± 12.5 eggs when using the hand-pressed method. A one-tailed *t*-test was performed to compare the differences between the ground truth and each method of preparation. In four of the ten samples, there was a significantly greater difference between the hand-pressed average and the gold standard compared to the difference between the stool-stomped average and the same gold standard. In the remaining samples, the Stool Stomper read average was closer to the gold standard in four samples, and none were statistically significant for the other method read. Furthermore, a Kolmogorov–Smirnov test found no significance between the two distributions at an alpha value of 0.05, indicating that the results were not attributable to abnormal distributions in either case. In summary, the Stool Stomper produces an average across all reads that is closer to the gold standard than the hand-pressed preparation method.

**Figure 7:**
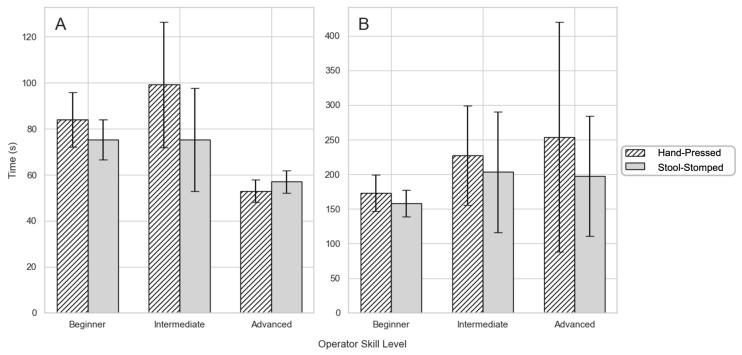
Analysis of Stool Stomper time savings separated according to lab technician experience levels. (**A**) Slide preparation time study comparing methods of preparation across technician skill levels. (**B**) Egg counting time study comparing methods of preparation across technician skill levels.

Figure 7 compares the differences in sample preparation and read time for both methods. Across nearly all technicians, stool-stomped samples had a lower variance of time to count/prepare as well as lower reading times on average. Figure 7A shows the time spent by each technician preparing microscope slides via the hand-pressed method or with the Stool Stomper device. Across all technicians, utilizing the Stool Stomper device reduced the average slide preparation time by 5.06 s (6.90%) compared to the hand-pressed method. On average, the hand-pressed slide preparation time was 73.31 ± 6.67 s, and the stool-stomped slide preparation time was 68.25 ± 4.88 s. When breaking down analysis by experience level, the beginner- and intermediate-level lab technician groups had decreased preparation time when using the Stool Stomper method, while the advanced-level group saw a slight increase in preparation time.

Figure 7B depicts the time spent reading the number of eggs for microscope slides prepared via the hand-pressed method or the Stool Stomper. In each of the three skill level classifications, i.e., beginner, intermediate, and advanced, the average time to count eggs across all reads was less when using the stool-stomped method when compared to the hand-pressed method. Across all technicians, the average egg counting time was reduced by 14.30 s (7.24%) by using the Stool Stomper device. On average, the hand-pressed egg counting time was 197.68 ± 25.34 s, while the stool-stomped egg counting time was 183.38 ± 18.36 s. Over the hundreds of slides read in the field each day, these time savings are substantial.

## 4. Discussion

One in five individuals worldwide is infected with soil-transmitted helminths [1,2,3]. Tanzania, an East African country that borders Lake Victoria, bears a significant burden from STH [20,21,22]. Although the prevalence of STH has decreased in some communities, the transmission rate indicates ongoing spread [23]. Thus, the accurate diagnosis, treatment, and prevention of soil-transmitted helminths and parasites remains a crucial public health issue worldwide, including in Tanzania.

To address this critical need, our study focused on creating a device to augment and improve the Kato–Katz protocol. Based on interviews with various stakeholders and field experts, we aimed to improve sample read time and standardize sample morphology. Lab technicians, who are the everyday users of the Kato–Katz protocol, emphasized the importance of the physical spread of the sample and the time to read the slides. These factors directly impact slide reading, and ultimately diagnosis and treatment, of soil-transmitted helminths. Higher reliability across technicians allows for more consistent diagnosis of STH infections at the community level to better inform treatment plans.

One well-known issue of the current Kato–Katz protocol is decreased reading acuity [24], leading to lower egg counts and the reduced administration of preventive chemotherapy to areas of need. When looking at egg count in Figure 5, statistical analysis supported that more eggs were found when reading the same stool sample prepared with the Stool Stomper. Therefore, a benefit of our device is addressing under-diagnosis [12,15], which would impact the distribution of preventative chemotherapy. Further analysis (Figure 6) across all reads for a given sample shows that the Stool Stomper produces an average that is closer to the gold standard than the hand-pressed preparation method.

Excitingly, the Stool Stomper showed reduced processing times for all technicians, with beginner/intermediate users showing the greatest improvement. According to prior reports, the standard Kato–Katz technique has an average preparation time of 8 min and 45 s per sample [25]. The Stool Stomper saved 3 min and 15 s per sample, where our results (Figure 7) indicate an average preparation time of 5 min and 45 s per sample. This advantage becomes more pronounced when compared to FLOTAC techniques, averaging over 20 min/sample [18,25]. Thus, the Stool Stomper results in significant workflow efficiency gains.

Another differentiating factor between these three different techniques is the price point. The reading costs, including salary, materials, and infrastructure, is USD 2.06 for duplicate Kato–Katz smears and USD 2.83 for a duplicate read using the FLOTAC technique [25]. The prototype Stool Stomper was created using 3D-printed ABS and PLA plastic for approximately USD 9. However, for wide-scale production, injection molding can be utilized, which we anticipate would lower device cost by at least 50%. Based on repeated, heavy device usage, we estimate a life span of approximately 5 years. With an average of 50 working days per year and 100 thick smear reads per day [25], the Stool Stomper has a per-read cost of 0.024 cents, and the projected cost-per-thick smear of a stool-stomped sample is USD 2.08. Despite these differences in cost, it is important to bear in mind that the salary of the lab technicians is one of the largest driving forces behind the varying costs of different methods of STH infection diagnosis [25,26,27]. Although our device costs more than the traditional Kato–Katz technique upfront, the Stool Stomper enables more rapid, standardized sample preparation from less experienced lab technicians.

The Stool Stomper design was further refined for scalable manufacturing. The current working prototype was constructed utilizing three separate 3D-printed plastic components and a steel rod to resist breakage upon falling at an angle. These components can, with relative ease, be mapped and converted to injection molds for rapid production. Ideally the entire system would be molded out of ABS plastic due to its structural integrity, light weight, and low price point. The steel rod is commercially available with a standard rod length and diameter. During the field testing conducted in this study, 12 devices were brought to Dodoma, Tanzania, following their manufacture in the United States. We anticipate that the Stool Stomper will be produced off-site, similar to the existing Kato–Katz kits which are not produced locally [6].

It is important to note that the prepared slides in this study were largely not subject to the decay exhibited in the field due to their refrigeration immediately following preparation and the nature of artificial eggs chosen. Furthermore, all technician reads of the same sample took place on the same day, reducing potential reading bias. Stool sample type and quality in this study varied greatly as ranked by the Bristol Stool Scale [28]. Most samples were Type 4—the most observed type in collected samples. However, Types 1 and 7 were also represented to ensure device reliability across a range of sample types, including the extremes.

One unanticipated challenge for the current study was the need for artificial eggs. Upon arrival at the testing site, the Ministry of Health informed the lab team that no stool samples infected with STH were available at the time, requiring a pivot in our intended device validation approach. Stool samples were spiked with shape-cut glitter as a substitute for the missing STH eggs. Advanced-level technicians and local field experts validated use of the artificial eggs as a proxy to continue with the study. Although the lack of access to real STH eggs is a limitation, our device was still validated with human stool samples to realistically assess sample preparation. In addition, the necessary egg substitution enables more controlled comparison by shape and color across methods of preparation for validation purposes. In summary, results from the current study show proof-of-principle, where findings motivate and inform future studies.

In summary, current results of the in-country field testing and pilot study are promising and support future Stool Stomper validation experiments in more extensive longitudinal studies. This study expands on the role of an innovative device in the Kato–Katz technique and demonstrates its advantages by analyzing intra-technician reading accuracy and time to prepare each slide. Specifically, design considerations and results indicate the following key advantages: (1) the Stool Stomper resulted in 14.30 s saved in egg counting per thick smear, and (2) higher reliability in egg counting was observed compared to the hand-pressed method. Although we project that production costs of the device will increase test cost by 0.02 cents across 5 years, net savings from reduced time and labor support implementation of the Stool Stomper. The results suggest that the standard Kato–Katz technique can be augmented to improve in-field accuracy and robustness, improving technician throughput and diagnostic potential. These benefits facilitate better distribution of preventive chemotherapy in high-STH-burden areas.

## Data Availability

Data are available upon reasonable request from the corresponding authors.

## Abbreviations

The following abbreviations are used in this manuscript:

ABS	Acrylonitrile Butadiene Styrene
NTD	Neglected Tropical Diseases
PLA	Polylactic Acid
STH	Soil-Transmitted Helminth
